# Cardiovascular manifestations in COVID-19 patients: A systematic review and meta-analysis

**DOI:** 10.34172/jcvtr.2021.30

**Published:** 2021-04-24

**Authors:** Seyyedmohammadsadeq Mirmoeeni, Amirhossein Azari Jafari, Seyedeh Zohreh Hashemi, Elham Angouraj Taghavi, Alireza Azani, Haniyeh Ghasrsaz, Azadeh Angouraj Taghavi, Seyed Hassan Niksima, Seyedyasin Rashidi, Erfan Kazemi, Hossein Sheibani, Seyed Sina Naghibi Irvani, Sahar Dalvand

**Affiliations:** ^1^Student Research Committee, School of Medicine, Shahroud University of Medical Sciences, Shahroud, Iran; ^2^Department of Pharmacology, Tehran University of Medical Sciences, Tehran, Iran; ^3^Department of Immunology, School of Medicine, Tehran University of Medical Sciences, Tehran, Iran; ^4^Department of Medical Genetics, Faculty of Medicine, Shahid Beheshti University of Medical Sciences, Tehran, Iran; ^5^Department of Biology, Science and Research Branch, Islamic Azad University, Tehran, Iran; ^6^Mazandaran University of Medical Sciences, Sari, Iran; ^7^School of Medicine, Tehran University of Medical Sciences, Tehran, Iran; ^8^Preventive Medicine and Public Health Research Center, Iran University of Medical Sciences, Tehran, Iran; ^9^Student Research Committee, Kashan University of Medical Sciences, Kashan, Iran; ^10^Clinical Research Developement Unit, Imam Hossein Hospital, Shahroud University of Medical Sciences, Shahroud, Iran; ^11^Research Institute for Endocrine Science, Shahid Beheshti University of Medical Sciences, Tehran, Iran; ^12^Functional Neurosurgery Research Center, Shohada Tajrish Comprehensive Neurosurgical Center of Excellence, Shahid Beheshti University of Medical Sciences, Tehran, Iran; ^13^Department of Epidemiology and Biostatistics, School of Public Health, Tehran University of Medical Sciences, Tehran, Iran

**Keywords:** Cardiovascular Disease, CVDs, COVID-19, SARS-CoV-2, Meta-Analysis

## Abstract

Since December 2019, the COVID-19 pandemic has affected the global population, and one of the major causes of mortality in infected patients is cardiovascular diseases (CVDs).For this systematic review and meta-analysis, we systematically searched Google Scholar, Scopus, PubMed, Web of Science, and Cochrane databases for all articles published by April 2, 2020. Observational studies (cohort and cross-sectional designs) were included in this meta-analysis if they reported at least one of the related cardiovascular symptoms or laboratory findings in COVID-19 patients. Furthermore, we did not use any language, age, diagnostic COVID-19 criteria, and hospitalization criteria restrictions. The following keywords alone or in combination with OR and AND operators were used for searching the literature: "Wuhan coronavirus", "COVID-19", "coronavirus disease 2019", "SARS-CoV-2", "2019 novel coronavirus" "cardiovascular disease", "CVD", "hypertension", "systolic pressure", "dyspnea", "hemoptysis", and "arrhythmia". Study characteristics, exposure history, laboratory findings, clinical manifestations, and comorbidities were extracted from the retrieved articles. Sixteen studies were selected which involved 4754 patients, including 2103 female and 2639 male patients. Among clinical cardiac manifestations, chest pain and arrhythmia were found to have the highest incidence proportion. In addition, elevated lactate dehydrogenase (LDH) and D-dimer levels were the most common cardiovascular laboratory findings. Finally, hypertension, chronic heart failure, and coronary heart disease were the most frequently reported comorbidities. The findings suggest that COVID-19 can cause various cardiovascular symptoms and laboratory findings. It is also worth noting that cardiovascular comorbidities like hypertension have a notable prevalence among COVID-19 patients.

## Introduction

On December 8, 2019, a cluster of acute respiratory illness, currently known as coronavirus disease 2019 (COVID-19) was discovered in Wuhan, China, the first sign of which was pneumonia.^1-6^ On March 11, 2020, the COVID-19 outbreak was considered as a pandemic health issue by the World Health Organization (WHO) Emergency Committee.^7^

Evidence shows that COVID-19 affects the myocardium; therefore, heart failure can be manifested in COVID-19 patients with cardiovascular diseases (CVDs).^4,8-10^ Also, cardiac injury is a common condition among hospitalized patients, which is linked with higher risk of mortality.^4^ Higher expression rates of angiotensin-converting enzyme 2 (ACE2) receptor in the heart and lungs of COVID-19 patients are suggested to be the reason for the cardiovascular manifestations in COVID-19 positive cases.^11-13^

It is worth mentioning that comorbidities like hypertension and CVD can cause a high case fatality rate in infected patients.^6,8,13^ In a study of 99 infected cases, 40% of the patients had a cardio-cerebrovascular disease.^14^ Accordingly, in an analysis of underlying diseases in 1099 confirmed patients, 15% of the patients were found to have hypertension and 27% had coronary heart disease (CHD).^15^ Moreover, it was suggested that cardiovascular comorbidities might promote the risk of mortality in COVID-19 patients.^8^

Considering the rapid spread of COVID-19, performing a meta-analysis with a large sample size to analyze the CVD manifestations, laboratory findings, and comorbidities in COVID-19 patients is urgently necessary. Therefore, this systematic review and meta-analysis is conducted to determine the rates of CVDs in COVID-19 patients based on the incidence proportion of cardiac manifestations, laboratory findings, and related comorbidities.

## Materials and Methods

### Data sources and searches

Five databases (i.e., Google Scholar, Scopus, PubMed, Web of Science, and Cochrane) were systematically searched (by S.M.) for all the articles published by April 2, 2020. The following MeSH-based keywords were used alone or in combination with OR and AND operators: “Wuhan coronavirus” OR “COVID-19” OR “coronavirus disease 2019” OR “SARS-CoV-2” OR “2019-nCoV” AND “cardiovascular disease” OR “CVD” OR “hypertension” OR “systolic pressure” OR “dyspnea” OR “hemoptysis” OR “arrhythmia”. In addition, the reference lists of the reviewed studies were scanned to identify other related articles to prevent missing data and to include all related studies. We used the Preferred Reporting Items for Systematic Reviews and Meta-Analyses (PRISMA) guidelines to report the information in this systematic review and meta-analysis.^16^

### Study selection

After a comprehensive systematic search, two of the authors (A.AJ. and S.M.) independently identified the eligible articles for review based on their titles and abstracts. Observational studies (cohort and cross-sectional designs) were included in this meta-analysis if they reported at least one of the related cardiovascular symptoms or laboratory findings in COVID-19 patients. Furthermore, we did not use any language, age, diagnostic COVID-19 criteria, and hospitalization criteria restrictions. Unpublished articles, interventional studies, systematic reviews, case reports, case series, commentaries, letters, correspondence articles, articles without full text, and other types of articles were excluded. Then, articles were selected for further full-text review by performing a careful screening by three of the authors (A.AT, E.AT, and E.K).

### Data extraction and quality assessment

The required data were independently extracted by three of the authors (i.e., A.A, SZ.H, and H.G), and disagreements or conflicts were resolved through discussion between three independent researchers (i.e., H.S, A.AJ, and S.M).

The following information was extracted and entered into an Excel spreadsheet: study characteristics (i.e., title of studies, author[s], year and month of publication, country name, sample size, study design, study sample characteristics [i.e., mean age, age range, gender, positive and negative patients, severe and non-severe patients, mortality, and survival]), exposure history (e.g., travel to Wuhan or contact with patients), clinical manifestations (fever, dry cough, expectoration, shortness of breath, muscle pain, headache, fatigue, sore throat, chills, snotty, diarrhea, dyspnea, nausea and vomiting, and gastrointestinal symptoms), laboratory findings (increased/decreased creatinine (Cr), increased D-dimer, increased/decreased blood urea nitrogen (BUN), positive-polymerase chain reaction (PCR) female, positive-PCR male, increased C-reactive protein (CRP), increased/decreased prothrombin time (Pt), increased lactate dehydrogenase (LDH), and increased/decreased creatine kinase (CK), and comorbidities (CHD, chronic heart failure (CHF), cerebrovascular disease, malignancy, hypertension, digestive system disease, pregnancy, hepatitis infection, diabetes mellitus, smoking, hyperlipidemia, endocrine disorders, chronic obstructive pulmonary disease (COPD), chronic respiratory disease, chronic kidney disease, and chronic liver disease).

The quality of the included studies was assessed by four independent researchers (A.AJ, S.M, SZ.H, and E.AT) based on the NIH quality assessment tool for observational cohort and cross-sectional studies.^17^ This instrument assesses the quality of included studies based on the research questions, study population, participation rate of eligible persons, inclusion and exclusion criteria, sample size justification, analyses, reasonable timeframe, exposure, outcome measures, outcome assessors, and loss to follow-up.

### Statistical analysis

Data from the included studies was extracted for the number of events and total patients to perform the meta-analysis using STATA statistical software, version 14 (Stata Corp). Cochran’s Q test and I^2^ index were used to examine the heterogeneity of the data. If the *P*-value for the Cochran’s Q test was below 0.1 (*P* <  0.1) or I^2^ index was above 50%, we used a random-effects model; otherwise, a fixed-effects model was used to estimate the pooled incidence proportion. Also, to stabilize the variances for each study, we adjusted the data by Freeman-Tukey double arcsine transformation and their 95% CIs were calculated by the Clopper-Pearson method.

We used some forest plots (for comprehensive visualization of the simply incidence point estimates) and the related CIs for each study along with summary measures.

## Results

### Study characteristics

**Figure 1:**
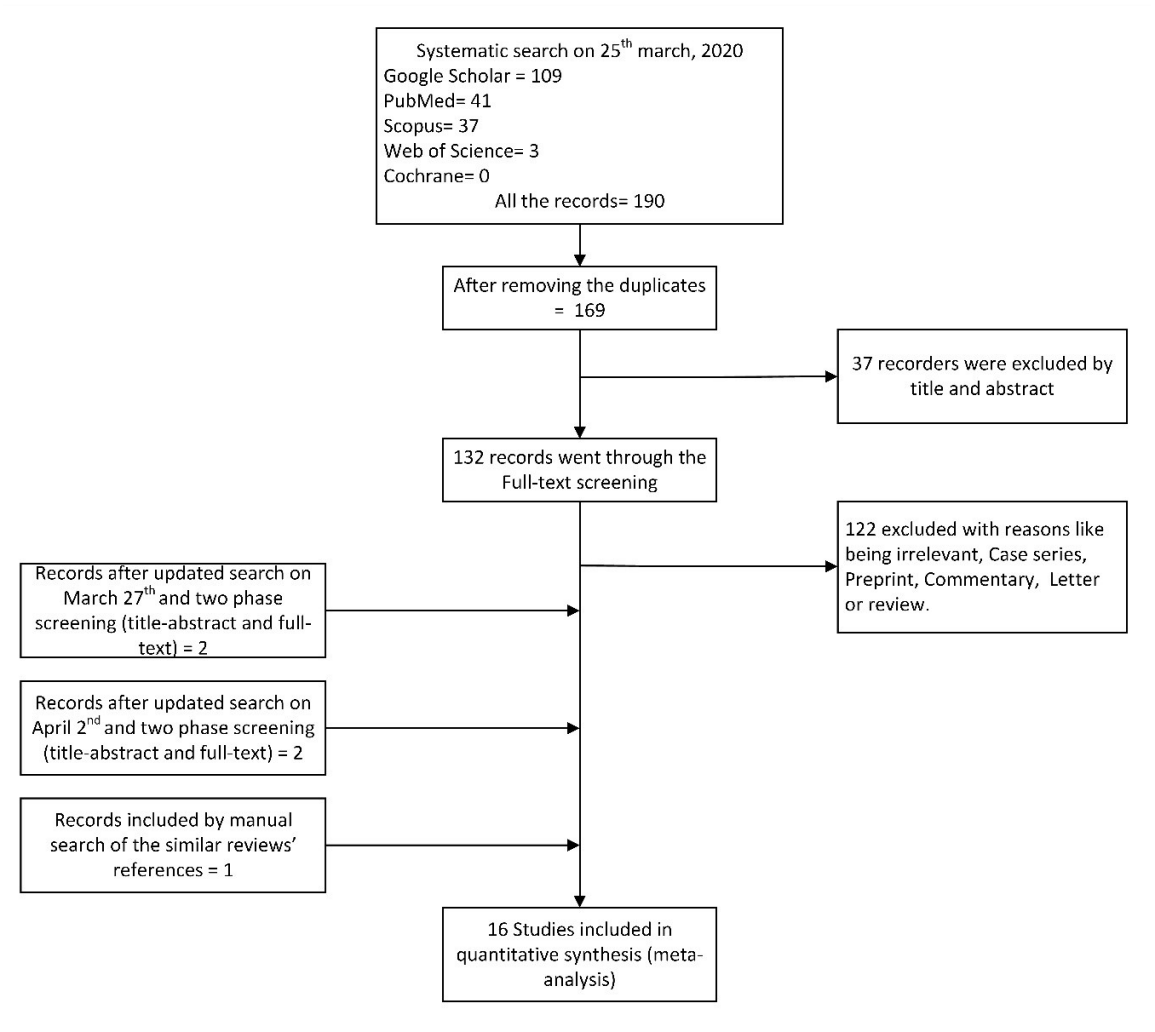
The process of surveying, screening, and selecting the articles for systematic review and meta-analysis based on PRISMA guideline

A flow diagram of our systematic search and the related screening processes is shown in Figure 1.

In our review, all the eligible published studies were conducted in China from January 1, 2020 to April 2, 2020. The total sample size of the 16 included studies presenting cardiovascular symptoms and laboratory results^4,5,8-10,14,15,18-26^ was 4754.

The largest and smallest study sample sizes belonged to the studies by Guan ^19^ with 1590 cases and Liu ^21^ with 30 participants, respectively. The main characteristics of our included studies are summarized in Table 1.

**Table 1:** Demographic and baseline characteristics of the included studies of COVID-19 patients presenting cardiovascular symptoms and comorbidities

**First author**	**Journal**	**Month of publication**	**City**	**Sample size(Male/female)**	**Mean age (Age range)**	**Quality assessment**	**Reference**
He, XW et al	Zhonghua xin xue guan bing za zhi	March	Wuhan	54(34/20)	68(-)	Fair	^20^
Chen, C et al	Zhonghua xin xue guan bing za zhi	March	Wuhan	150(84/66)	59(14-96)	Fair	^18^
Chen, N et al	The Lancet	January	Wuhan	99(67/32)	55.5(21-82)	Good	^14^
Li, K et al	Invest Radiol	February	Not Determined	83(44/39)	45.5(-)	Good	^9^
Huang, C et al	The Lancet	January	Wuhan	41(11/30)	49(-)	Good	^8^
Liu, K et al	Chin Med J (Engl)	January	Not Determined	137(61/76)	57(20-83)	Fair	^10^
Liu, M et al	Zhonghua Jie He He Hu Xi Za Zhi	February	Wuhan	30(10/20)	35(21-59)	Fair	^21^
Peng, YD et al	Zhonghua Xin Xue Guan Bing Za Zhi	February	Wuhan	112(53/59)	62(55-67)	Good	^22^
Shi, S et al	JAMA Cardiol	March	Wuhan	416(205-211)	64(21-95)	Good	^4^
Tian, S et al	Journal of Infection	February	Beijing	262(127-135)	47.5(1-94)	Fair	^23^
Wu, C et al	JAMA Intern Med	March	Wuhan	201(128/73)	51(43-60)	Good	^5^
Yang, W et al	J Infect	February	Wenzhou	149(81/68)	45.1(-)	Fair	^24^
Zhang, JJ et al	Allergy	February	Wuhan	140(71/69)	57(-)	Good	^25^
Zhou, F et al	Lancet	February	Wuhan	191(119/72)	56(-)	Good	^26^
Guan, WJ et al	Eur Respir J	March	Not Determined	1590(904/674)	48.9(-)	Fair	^19^
Guan, Wei-j et al	New England Journal of Medicine	April	Wuhan	1099(640-459)	47(35-58)	Good	^15^

### Epidemiological characteristics

Based on the random-effects model, the rates of patient survival and mortality (Table 2) were 0.8571 (95% CI, 0.7536-0.9365) and 0.1056 (95% CI, 0.0559-0.1681), respectively.

**Table 2:** Mortality, survival, and the exposure history of COVID-19 patients with cardiovascular symptoms or comorbidities

**Variable**	**No studies**	**Total sample size**	**No positive case**	**Incidence rate** **(95% CI)**	**Heterogeneity**		
					**I** ^2^ ** (%)**	**Q**	***P*** ** value**
Mortality	13	4501	310	0.1056 (0.0559-0.1681)	96.8	378.6	< 0.0001
Survival	11	4150	3813	0.8571 (0.7536-0.9365)	98.3	599.8	< 0.0001
Exposure History							
Imported	4	692	381	0.4817 (0.0546-0.9089)	99.5	665.9	< 0.0001
Travel to Wuhan	6	3240	1793	0.5231 (0.1958-0.8504)	99.7	2076.1	< 0.0001
Contact with patients	2	292	159	0.5666 (0.5048-0.6238)	-	-	-

In addition, the pooled incidence proportion of exposure history of traveling to Wuhan and Huanan seafood market was 0.5231 (95% CI, 0.1958-0.8504). Further information as to the epidemiological characteristics is provided in Table 2.

### Clinical manifestations and laboratory finding

**Figure 2:**
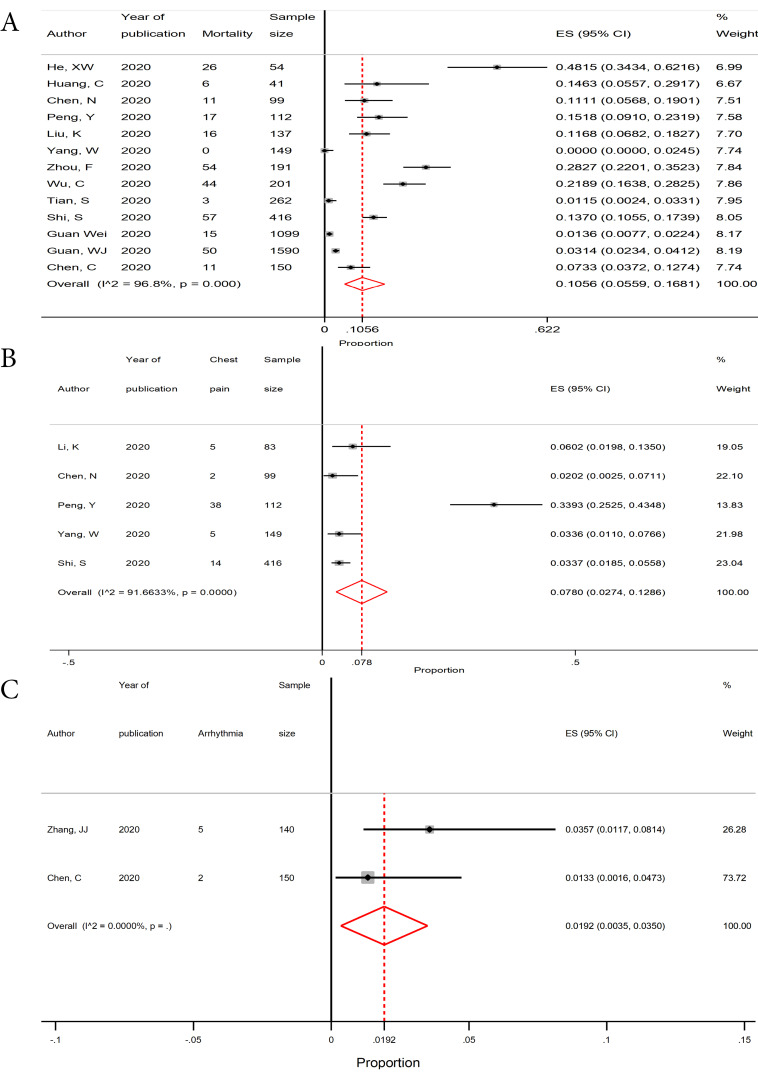
Forest plot of the incidence proportion of mortality (A), chest pain (B), and arrhythmia(C) in COVID-19 patients presenting cardiovascular manifestations or comorbidities

Chest pain and arrhythmia with the incidence proportions of 0.0780 (95% CI, 0.0274-0.1286) and 0.0192 (95% CI, 0.0035-0.0350) were the most common cardiac clinical manifestations (Figure 2). Among non-cardiac manifestations, fever (0.7986, 95% CI, 0.7103-0.8869), dry cough (0.6381, 95% CI, 0.5635-0.7126), and fatigue (0.3927, 95% CI,.3092-0.4761) were the most frequently observed clinical manifestations (Table 3).

**Table 3:** Clinical manifestations of COVID-19 patients presenting cardiovascular symptoms

**Variable**	**No studies**	**Total sample size**	**No positive case**	**Incidence rate** **(95% CI)**	**Heterogeneity**		
					**I** ^2^ ** (%)**	**Q**	***P*** ** value**
Cardiac manifestations							
Chest pain	5	859	64	0.0780 (0.0274-0.1286)	91.6	47.9	< 0.0001
Arrhythmia	2	290	7	0.0192 (0.0035-0.0350)	-	-	-
other manifestations							
Fever	15	4604	3414	0.7986 (0.7103-0.8869)	98.4	878.3	< 0.0001
Dry cough	15	4604	2910	0.6381 (0.5635-0.7126)	96.2	367.9	< 0.0001
Expectoration	6	1177	219	0.2046 (0.0960-0.3131)	96.9	169.9	< 0.0001
Shortness of breath	6	3398	738	0.2405 (0.2006-0.2805)	86.1	26.4	0.0001
Muscle pain	7	2091	251	0.1118 (0.0625-0.1612)	92.1	75.9	< 0.0001
Headache	10	3906	443	0.1051 (0.0638-0.1464)	94.3	157.3	< 0.0001
Fatigue	11	4219	1480	0.3927 (0.3092-0.4761)	96.7	306.1	< 0.0001
Sore throat	6	3436	391	0.0921 (0.0454-0.1388)	94.9	99.7	< 0.0001
Chills	3	2838	310	0.1086 (0.0972-0.1200)	-	-	-^*^
Snotty	3	664	19	0.0275 (0.0150-0.0399)	-	-	-^*^
Diarrhea	11	4028	183	0.0471 (0.0331-0.0610)	70.4	33.8	0.0002
Dyspnea	7	903	171	0.2340 (0.1275-0.3405)	96.9	194.6	< 0.0001
Nausea and vomiting	6	3268	170	0.0450 (0.0247-0.0653)	84.3	31.8	< 0.0001
Gastrointestinal symptoms	3	253	71	0.2561 (0.0296-0.4826)	94.7	37.6	< 0.0001

^*^Fixed effects model

**Figure 3:**
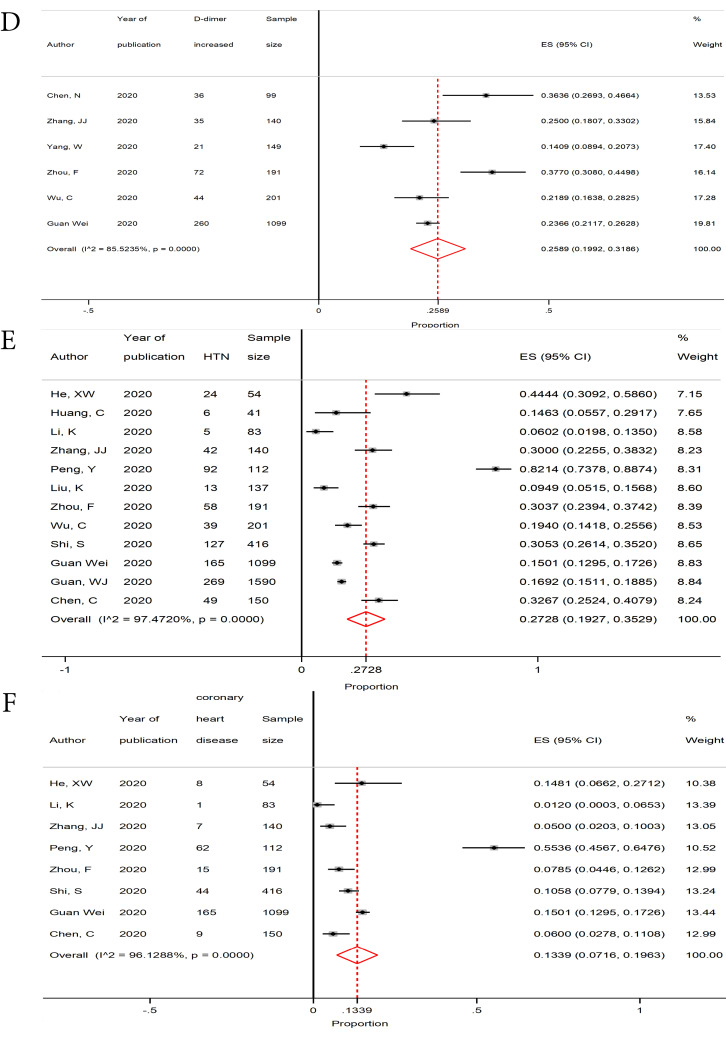
Forest plot of the incidence proportion of D-dimer (D), hypertension (E), and chronic heart disease (F) in COVID-19 patients presenting cardiovascular manifestations or comorbidities

Moreover, among all cardiovascular variables, elevated lactate dehydrogenase (LDH) (0.5422, 95% CI, 0.3546-0.7298) and D-dimer (0.2589, 95% CI, 0.1992-0.3186) levels were the most commonly reported clinical findings (Table 3, Figure 3).

The results regarding clinical manifestations and laboratory findings are presented in Table 3 and Table 4, respectively.

**Table 4:** Cardiovascular laboratory findings in COVID-19 patients

**Variable**	**No studies**	**Total sample size**	**No positive case**	**Incidence rate (95% CI)**	**Heterogeneity**		
					**I** ^2^ **(%)**	**Q**	***P*** ** value**
Increased Cr	6	1780	79	0.0722 (0.0286-0.1157)	92.6	67.4	< 0.0001
Decreased Cr	3	289	59	0.3723 (0.000-0.8646)	99.5	386.2	< 0.0001
Increased Pt	4	640	37	0.0559 (0.0193-0.0924)	77.9	13.6	0.0036
Decreased Pt	2	248	34	0.0478 (0.0229-0.0728)	-	-	-
Increased CK	5	1678	141	0.0815 (0.0494-0.1136)	77.0	17.4	0.0016
Decreased CK	2	248	42	0.1582 (0.1132-0.2033)	-	-	-
Increased BUN	2	300	15	0.1326 (0.0905-0.1746)	-	-	-
Decreased BUN	2	248	34	0.6712 (0.4735-0.8688)	-	-	-
Positive PCR female	7	1236	613	0.5243 (0.4532-0.5953)	82.5	34.3	< 0.0001
Positive PCR male	7	1236	623	0.4757 (0.4047-0.5468)	82.5	34.3	< 0.0001
Increased D dimer	6	1879	468	0.2589 (0.1992-0.3186)	85.5	34.5	< 0.0001
Increased LDH	4	640	337	0.5422 (0.3546-0.7298)	96.2	78.6	< 0.0001
Increased CRP (> 10 mg/L)	6	1624	850	0.6712 (0.4735-0.8688)	98.3	304.4	< 0.0001

Abbreviations: BUN, blood urea nitrogen; Cr, creatinine; CRP, C-reactive protein; Pt, prothrombin time; CK, creatine kinase; PCR, polymerase chain reaction; LDH, lactate dehydrogenase

### Comorbidities

**Figure 4:**
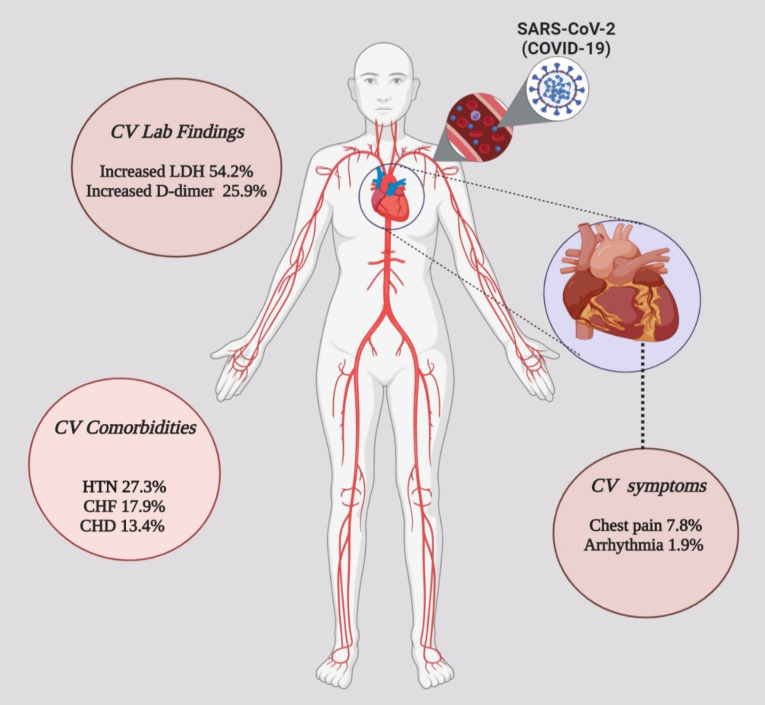
Cardiac manifestations, lab findings and comorbidities with the highest incident rates in the COVID-19 patients. (this figure is Created with BioRender.com)

According to our results, the pooled prevalence of hypertension was 0.2728 (95% CI, 0.1927-0.3529) in 12 studies. Also, CHF and CHD with 0.1788 (95% CI, 0.000-0.3824) and 0.1339 (95% CI, 0.0716-0.1963) had the highest prevalence after hypertension (Table 5, Figure 4).

**Table 5:** Cardiovascular comorbidities in COVID-19 patients

**Variable**	**No studies**	**Total sample size**	**No positive case**	**Incidence rate (95% CI)**	**Heterogeneity**		
					**I** ^2^ ** (%)**	**Q**	***P*** ** value**
Cardiac comorbidities							
Coronary heart disease	8	2245	311	0.1339 (0.0716-0.1963)	96.1	180.8	< 0.0001
Chronic heart failure	3	569	63	0.1788 (0.000-0.3824)	95.9	49.3	< 0.0001
Hypertension	12	4214	889	0.2728 (0.1927-0.3529)	97.5	435.1	< 0.0001
Non-cardiac comorbidities							
Chronic Respiratory Disease	4	589	9	0.0118 (0.0031-0.0206)	-	-	-^*^
Chronic kidney disease	7	3787	302	0.0397 (0.0041-0.0752)	97.9	283.1	< 0.0001
Chronic liver disease	3	382	22	0.0503 (0.0100-0.0905)	66.9	6.0	0.0487
Cerebrovascular disease	6	797	77	0.0218 (0.0095-0.0382)	81.8	27.5	< 0.0001
Malignancy	12	4267	169	0.0231 (0.0091-0.0372)	89.7	107.2	< 0.0001
Digestive system disease	3	388	26	0.0120 (0.0030-0.0210)	-	-	-^*^
Pregnancy	2	566	8	0.0120 (0.0030-0.0210)	-	-	-
Hepatitis Infection	3	3105	51	0.0155 (0.0111-0.0198)	-	-	-^*^
Diabetes mellites	12	4214	431	0.1245 (0.1003-0.1488)	78.3	50.7	< 0.0001
Smoking	5	3061	271	0.0801 (0.0495-0.1108)	83.9	24.6	0.0001
Hyperlipidemia	2	194	11	0.0551 (0.0230-0.0871)	-	-	-
Endocrinology disorders	4	589	29	0.0498 (0.0107-0.0809)	83.3	17.9	0.0004
COPD	9	3751	66	0.0150 (0.0111-0.0189)	-	-	-^*^

Abbreviatins: COPD, chronic obstructive pulmonary disease ^*^Fixed effects model

## Discussion

The COVID-19 outbreak has become a major public health issue around the world.^27^ In addition to the devastating respiratory outcomes of COVID-19, the impact of this disease on the cardiovascular system is notable.^20^ As mentioned earlier, in this systematic review and meta-analysis, we attempted to focus specifically on the cardiovascular manifestations and related comorbidities of COVID-19 to underscore the fact that the cardiovascular aspect of COVID-19 is as important as the respiratory complications.

In this study, the mortality rate of COVID-19 patients presenting cardiovascular manifestations or related laboratory findings was 10.6%. A recent study performed among COVID-19 patients suggested that in-hospital mortality in patients with myocardial injury was higher than that in other patients.^28^ Also, it has been reported that cardiac dysfunction and myocardial injury can occur in approximately 20% of COVID-19 patients.^18^ Despite that the mechanism of this injury is not completely clear, cytokine storm and direct viral damage to myocardial cells are assumed to be the underlying reasons for such incidents in COVID-19 patients.^29-31^

According to our findings, the most common symptoms in COVID-19 patients were fever (79.8%), dry cough (63.8%), fatigue (39.2%), shortness of breath (24%), and dyspnea (23.4%). This result is consistent with the reports of a recent meta-analysis on COVID-19.^32^ Recent studies have shown that some COVID-19 patients present severe cardiovascular manifestations such as acute myocarditis and heart failure.^4,6,8,33,34^

We found that the incidence proportion of chest pain and arrhythmia in COVID-19 patients were 7.8% and 1.9%, respectively. Additionally, based on a recent study cardiac symptom like chest tightness, chest pain, and arrhythmia were more common among old, hospitalized, and severe COVID-19 patients.^4^ Wei et al,^35^ also found that severe myocardial injury can affect the prognosis of COVID-19. Recent investigations have revealed that SARS-CoV-2 spike protein can bind to the ACE2 receptor.^12^ ACE2 is a membrane-bound aminopeptidase that is highly expressed in the heart and lungs.^11^ Therefore, it is suggested that SARS-CoV-2 mainly invades alveolar epithelial cells and the myocardium, resulting in respiratory and cardiovascular symptoms like dyspnea, chest pain, and arrhythmia.^11^ Accordingly, the mechanism of acute myocardial injury in COVID-19 might be related to ACE2.^11^

Laboratory findings revealed that elevated D-dimer (25.8%) and LDH (54.2%) levels were the most common cardiovascular clinical results. In accordance with our results, a systematic review and meta-analysis performed by Fu et al on Chinese patients with COVID-19 indicated that the incidence proportion of increased D-dimer was 29.3%.^32^ Since elevated D-dimer is an independent risk factor for CVD events, it can predict the short- and long-term risks of CVD mortality.^36,37^ Increased levels of high-sensitivity cardiac troponin I (cTnI) along with other inflammatory biomarkers like D-dimer can be a possible reason for myocardial injury in COVID-19 patients.^13^

It is suggested that LDH has a high accuracy in the prediction and early recognition of COVID-19 cases.^38,39^ Based on Kopel et al LDH has an independent association with CAD.^40^ In myocardial ischemia, the elevated serum level of LDH is a useful, but not-specific, diagnostic biomarker for acute myocardial infarction.^41^ Besides, maintaining the serum LDH level within the normal range can lower the risk of atherosclerotic CVDs, and it could be a valuable biomarker for assessing the risk of CVDs.^42^

HTN is the main risk factor for CVDs and it is associated with several cardiac problems like CHD and heart failure.^43^ In this regard, it is necessary to note that in our study, hypertension (27.2%) was the most important cardiovascular comorbidity in COVID-19 patients. It is reported that among the patients with severe symptoms of COVID-19, 58% of them had hypertension and 25% had heart disease. Moreover, CHD (13.3%) and CHF (17.8%) were other critical cardiovascular comorbidities in our study. Furthermore, based on the National Health Commission report of China, 17% of the patients diagnosed with COVID-19 had CHD.^44^

As shown in Figure 4, we suggest that COVID-19 can have cardiovascular manifestations such as chest pain and arrhythmia along with elevated serum D-dimer and LDH levels. On the other hand, increased levels of D-dimer and LDH can be an additional risk factor for CVD in COVID-19 patients. Thus, they could be considered as an additional diagnostic tool and therapeutic opportunity in COVID-19 patients. Also, hypertension, CHD, and CHF are the major cardiovascular comorbidities in COVID-19 patients.

One of our limitations is that due to the new pandemic COVID-19, there were a few studies that met our inclusion criteria, so we could not measure other cardiovascular paraclinical tests like electrocardiography and echocardiogram. Because of some lack of information, the results would not be applicable to all covid-19 patients.

## Conclusion

In a nutshell, it is possible that cardiovascular manifestations and their relevant laboratory findings could have a notable effect on the COVID-19 patients’ outcomes, but future investigations should be performed to enlighten the cardiovascular aspects of COVID-19.

## Competing interest

None declared.
